# Influence of Geometric Properties of Capacitive Sensors on Slope Error and Nonlinearity of Displacement Measurements

**DOI:** 10.3390/s21134270

**Published:** 2021-06-22

**Authors:** Lars Daul, Tao Jin, Ingo Busch, Ludger Koenders

**Affiliations:** 1 Physikalisch-Technische Bundesanstalt (PTB), Bundesallee 100, 38116 Braunschweig, Germany; Ingo.Busch@ptb.de (I.B.); ludger.koenders@ptb.de (L.K.); 2 School of Optical-Electrical and Computer Engineering, University of Shanghai for Science and Technology (USST), Yangpu District, Shanghai 200093, China; jintao@usst.edu.cn

**Keywords:** capacitive sensors, displacement measurements, slope error, nonlinearity, FEM simulation

## Abstract

Capacitive sensors are widely used in industrial applications, such as CNC machine tools, where reliable positioning in the micrometer range with nanometer accuracy is required. Hence, these sensors are operated in harsh industrial environments. The accuracy of these sensors is mainly limited by slope errors and nonlinearities. In practice, the required accuracy of these sensors is achieved by a calibration against a metrological high-quality reference such as interferometric displacement measurement systems. This usually involves the use of high-order polynomials as calibration functions based on empirical data. In metrology, this is only the second-best approach and has disadvantages in terms of stability over the measurement range of the instrument. In addition, the validity of these empirical calibrations over time is questionable, and the associated uncertainty can only be roughly estimated. This makes regular recalibration of such sensors at short intervals mandatory to ensure the reliability of the displacement measurement. In this paper, we report on our investigations of the different parameters that affect the accuracy of capacitive sensors. Since the capacitance of these sensors results from the electric fields that build up between the electrodes, these field lines are calculated using FEM simulation models for typical commercial sensors. In the following the influence of various geometric parameters such as edge radius, guard ring size and shape, or thickness of the electrodes are individually analyzed according to their impact on the accuracy of these sensors. Based on these simulations, the deviations of the capacitance as they arise for real detector geometries can then be compared with idealized, de facto unrealizable parallel plate capacitors. This methodology allows overall uncertainty of capacitive sensors to be decomposed into their individual components and sorted in terms of their contribution to the uncertainty budget. The individual FEM-based analysis then enables a systematic analysis of the sources of uncertainty and, thus, reveals possibilities to improve manufacturing processes for capacitive sensors, to put these sensors on a solid metrological basis, and to improve the performance of these displacement measurement systems in the long run, i.e., to provide better sensors for the application.

## 1. Introduction

Nowadays, the primary method for practical realization of the meter is based on direct or indirect measurement of the time of flight of light [1]. Nevertheless, capacitive sensors have long been an integral part of dimensional measurement technology [2,3]. They are used for small-range (e.g., below 100 µm) noncontact displacement measurements [4,5]. Compared to laser interferometers, they are compact and can, therefore, be used in many industrial and metrological applications such as ultra-high precision manufacturing [6] and ultra-precise positioning systems [7,8,9]. Their resolution reached the single-digit nanometer range [10]. However, capacitive sensors are not mentioned in the definition of the meter, as they do not fulfill the metrological requirements. For this reason, we want to investigate capacitive sensors in more detail in order to gain a better understanding of the causes of measurement deviations.

In capacitive displacement measurements, plate and cylindrical capacitors are used depending on their range and application. One fixed electrode (probe) detects the displacement of the second electrode (target), which contacts the item to be moved. If this item is electrically conductive and smaller than the area of the probe electrode, the target can be removed and a contactless measurement is possible [11]. For measurements with high sensitivity but small displacement, the target is moved perpendicular to the probe surface, which corresponds to a displacement out of plane. In the ideal case of an infinitely extended parallel plate capacitor, the inverse capacitance changes linearly with the distance between the electrodes. The ideal Equation (1) can be used:

(1) $$C=\frac{\varepsilon_{0}\varepsilon_{r}A_{geom}}{d}$$

with the capacitance C, the permittivity of vacuum $\varepsilon_{0}$, the relative permittivity of the material between the plates $\varepsilon_{r}$, the geometric surface area of the probe electrode $A_{geom}$, and the distance between the plates d.

Using this equation, the displacement can be converted into an electric signal. However, Equation (1) is only valid if there are no stray capacitances from the side and top of the electrodes and no distortions of the electric field. These fringe fields occur, for example, at the edges of the electrodes. They are the reason for deviations from an ideal case and, thus, result in a slope error and nonlinearities. These edge effects can be reduced by using a guard ring according to the principle of Lord Kelvin and Clerk Maxwell [12]

In this study, the capacitive sensor is defined as a combination of a working electrode, which is the part of the capacitive sensor whose capacitance is used for displacement measurement, and an active shield, the guard ring, surrounding it. Both have the same electric potential and are electrically separated by a dielectric. Thus, the working electrode is not only passively protected from external interference signals, but its electric field is actively homogenized and nonlinearity is reduced [9]

Many influencing parameters on nonlinearity were investigated in previous studies. Among these are:

- Environment (temperature, humidity) [8];
- Adjustment (tilt) [9];
- Geometric parameters (dielectric gap between the working electrode and the guard ring) [13].

With increasing demands on linear capacitive displacement measurements, the influence of the sensor surface quality has become increasingly important, starting with the surface roughness [14,15]. In addition, real surfaces are not equipotential surfaces [16]. Local differences of the chemical composition in metal alloys, surface adsorption, grain boundaries in polycrystalline structures, and contamination cause local variations of the work function on the sensor surface. These lead to regions with different surface potential [17]. This is known as the patch effect [18]. In order to investigate whether such patch potentials also influence the nonlinearity of capacitive displacement sensors, all other influencing variables must be known. Therefore, the influences of geometric properties are studied in this work.

For practical applications, deviations from an ideal displacement measurement are determined by a calibration [5] and compensated electronically [19]. The general objective of this project is the systematic analysis of the different errors of capacitive displacement sensors in order to gain a comprehensive overview of the uncertainties and to compare them with the primary method for realizing the meter, the interferometry. This will be the basis for reliably estimating the magnitude of the applied calibration factor, which should be as close to one as possible from the metrological point of view.

This study is the starting point of such a detailed uncertainty analysis, which is required to identify options to improve the capacitive displacement sensor in the future and to increase their metrological reliability. Using the finite element method (FEM) to simulate the geometry of real capacitive sensors, the simulated capacitance of a parallel plate capacitor is compared with that of an ideal parallel plate capacitor and a capacitive sensor.

Based on the progression of the electric field lines, the difference between the three capacitances is discussed. The results are used to explain slope error as a consequence of the difference between the geometric and the active area of the sensor, as well as stray capacitances. Analogously, the nonlinearity can be physically described by the different disturbances of the electric field lines.

In a parameter study, the following influencing parameters on the capacitance, the slope error, and the nonlinearity are calculated and quantitatively compared: the width of the dielectric gap between the probe and its guard ring; the edge radius of the probe and the guard ring; the thickness of the probe; the width of the guard ring. The dependence of the influence of the displacement range and of the starting distance is also simulated. The results of the FEM analysis will be used to design an experiment to verify the simulation model and to calculate an uncertainty budget.

## 2. FEM Simulation of the Capacitance of Parallel Plate Capacitor

The multitude of influencing parameters makes the experimental analysis of an individual parameter difficult. Maxwell calculated the electrical capacitance of a parallel plate capacitor with a guard ring electrode using conformal transformation [20]. However, this solution leads to restrictions in the dimensions of the electrodes. A complete analytical solution is possible by solving the Laplace equation, but only for a few simplified problems [12]. In recent studies, it has been shown that simulations with the FEM are an appropriate technique to investigate individual sensor properties of real capacitive sensor geometries [21,22].

**Figure 1:**
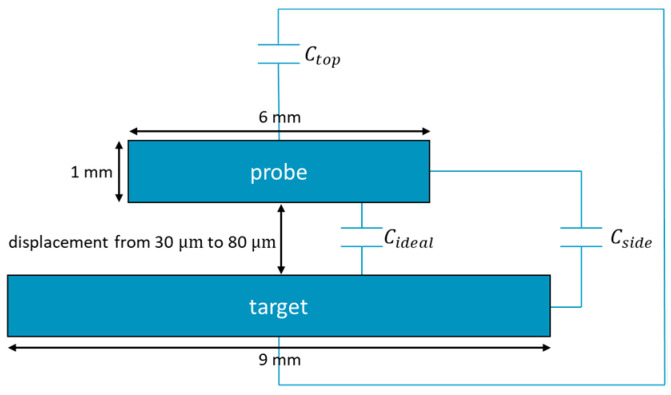
Sketch of the geometry of the initial model. Two discs form a parallel plate capacitor. The dimensions correspond to the geometry of real capacitive sensors. In addition to the capacitance of ideal parallel plate capacitor, the top and side also contribute to the total capacitance.

The COMSOL Multiphysics software (version 5.4) is used for the FEM simulations. There is a large disc with a radius of 4.5 mm, and there is a small disc with a radius of 3 mm. Their thickness is 1 mm. The software requires the specification of material parameters for the simulation, even if they are not used for the calculation of the capacitance. Thus, aluminum 6063-T83 is added to both cylinders. The material and geometry used in the model correspond to the material and geometry of real, commercially available sensors, such as those provided by Micro-Epsilon or PI. The geometry is illustrated in Figure 1.

The mechanical, thermal, and electric properties of the material are contained in the software database. Dirichlet boundary conditions are defined, according to which the electric potential on the top cylinder is 1 V and on the bottom cylinder, 0 V [23]. The electric potential difference generates an electric field. The two cylinders form a parallel plate capacitor with the capacitance $C=\frac{Q}{V}$ with the electric potential difference *V*, and the stored electric charge in the capacitor *Q*. At a separation *d* = 30 µm, the capacitance of the ideal parallel plate capacitor is

(2) $$C_{ideal}=\frac{\varepsilon_{0}\varepsilon_{r}A_{geom}}{d}=\frac{1\times 8.854\;\frac{\mathrm{As}}{\mathrm{Vm}}\times \pi \times {\left(3\times 10^{-3}m\right)}^{2}}{30\times 10^{-6}m}=8.34\times 10^{-12}\frac{\mathrm{As}}{V}=8.34\;\mathrm{pF}$$

with the capacitance C, the permittivity of vacuum $\varepsilon_{0}$, the relative permittivity of the material between the plates $\varepsilon_{r}$, and the distance between the plates d. The geometric surface area of the probe electrode $A_{geom}$ is used for the calculation, since the distortion of the electric field lines due to the edges is not considered in the ideal equation, and the electric field lines do not extend beyond the geometric surface.

For the FEM simulation of the system, a sphere with the material air is created as the surroundings. The electric field of the plates can, thus, propagate in space. Only the course of the electric field lines in the vicinity of the plates is relevant for this study. To reduce the influence of the virtual boundary of the environment, spherical infinite boundary conditions are used for the air sphere: the edge of the surrounding sphere is divided into two layers. The solving algorithm assumes the electric field between these layers to be constant, and the dimension of the sphere is virtually increased by a factor of 1000. As a result, the size of the model can be reduced to the significant area, while also minimizing the influence of the artificial boundaries of the model on the simulation result.

Near the electrodes, the model is divided into tetrahedra by the COMSOL Multiphysics algorithm. The smallest possible mesh size is chosen such that the simulation result converges, and a change in mesh size no longer has any influence on the simulation result.

**Figure 2:**
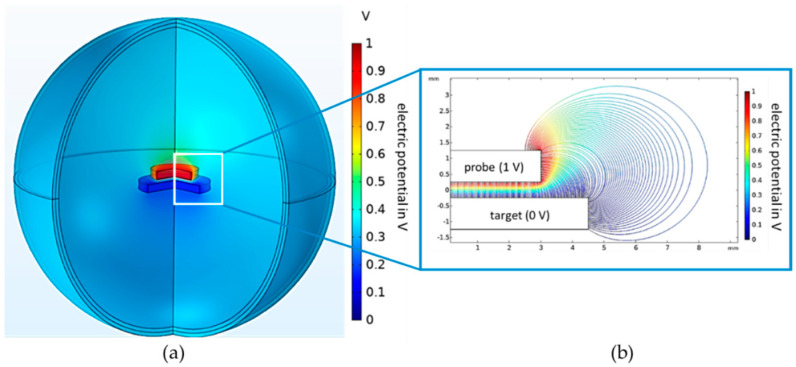
FEM simulation result of the parallel plate capacitor. (**a**) Electric potential distribution of the complete model. The probe has an electric potential of 1 V and the target of 0 V. The surroundings are an air sphere with infinite boundary conditions. (**b**) Propagation of the electric field lines. There are fringe fields at the edges of the electrodes.

Gauss’ law of electrostatics is solved for the electric field. The scalar electric potential is the dependent variable. The computing time is reduced by exploiting the rotational symmetry of the model. Gauss’ law of electrostatics is only calculated for half of a two-dimensional section. Using rotationally symmetric boundary conditions, the two-dimensional simulation result is rotated by 360°. This results in the three-dimensional solution of the electric field distribution, presented in Figure 2.

The three-dimensional solution is shown in the left graphic. The colors correspond to the electric potential distribution. The top electrode (probe) has an electric potential of 1 V, and the bottom electrode (target) has an electric potential of 0 V. The electric field propagates in the air sphere.

To compute the capacitance of the plate capacitor, the electric charge on the plates is divided by the electric potential. For this model with a plate distance of *d* = 30 µm, the calculated capacitance is

(3) $$C_{simul}=\frac{Q}{V}=8.97\;\mathrm{pF}.$$

The influence of numeric errors of the program, such as rounding errors, can be neglected in the ongoing process. For the described model, the reproducibility is estimated by $2\times 10^{-12}$ pF.

The simulated capacitance is 7.6% larger than the capacitance of ideal parallel plate capacitor. The progression of the field lines in space causes this deviation between the two capacitances: the electric field lines extend beyond the geometric surface of the electrodes. Thus, there is an active area, which is larger than the geometric area. In addition, electric field lines also radiate from the sides and top of the probe, which lead to the parasitic capacitances $C_{top}$ and $C_{side}$ [24]. This results in the difference between the ideal and the simulated capacitance.

## 3. FEM Simulation of a Capacitive Displacement Measurement

The capacitances for 10 plate distances in the range from 30 to 80 µm are calculated to simulate a capacitive displacement measurement. For the conversion of the simulated capacitances into plate distances, the ideal formula (Equation (1)) with the geometric probe surface area is used. This leads to an error. However, this error is constant and, therefore, does not affect the investigation of the influence of the geometrical parameters on the capacitive displacement measurement.

**Figure 3:**
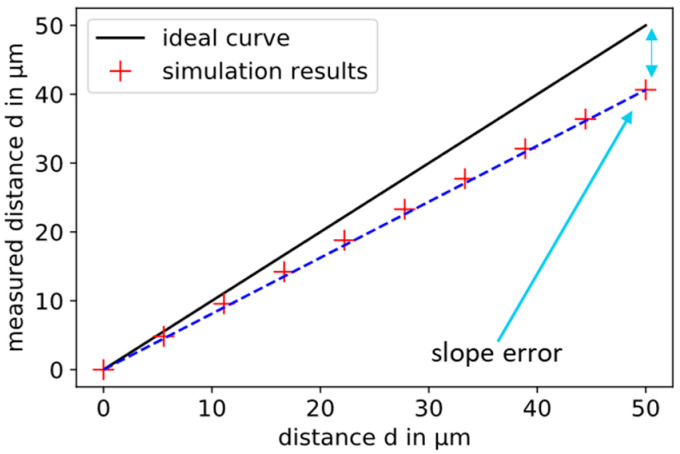
Result of the simulated capacitive displacement measurement with a simple parallel plate capacitor. The black line corresponds to the ideal curve. The red crosses are the simulation results. The capacitance is calculated for 10 plate distances and converted into a “measured” displacement using the ideal Equation (1). The minimum and the maximum are connected by a straight line (dashed blue). The difference between the end of the ideal line and the last calculated displacement is the slope error.

In Figure 3, the calculated “measured” distances (red crosses) are compared to the ideal displacement curve (black line). The *x*-axis and the *y*-axis are normalized. This results in the simulation of a relative displacement measurement of 50 µm.

The calculated distances are always smaller than the ideal distances, because the smaller known geometric area is used for the conversion, and stray capacitances are not taken into account. Thus, only a displacement of 40.65 µm can be “measured” instead of the 50 µm. This difference is called the slope error. It is connected to the sensitivity of the inverse of the capacitance related to the change in the plate distance. If the sensitivity increases, the slope error becomes smaller. In the simulation presented, the slope error is 9.35 µm. When applied practically, this error is typically corrected by a suitable calibration. In this work, the influence of the geometric parameters on the slope error is investigated in order to keep the calibration factor as small as possible by a suitable choice of geometry.

**Figure 4:**
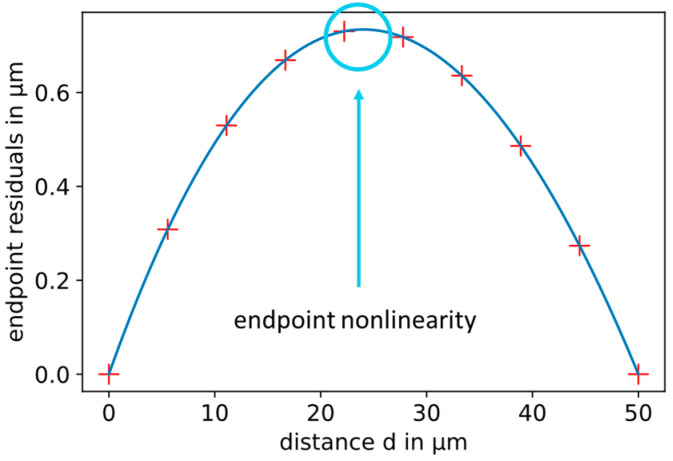
Residuals from the straight line (red crosses). The maximum residual is the endpoint nonlinearity, which is calculated by a fit (blue curve).

To compare the nonlinearities of different sensor setups, the following method is used. The smallest calculated distance is connected to the largest calculated distance by a straight line (dashed blue line in Figure 3). The differences of the simulated points to this line are plotted as residuals over the displacement according to Figure 4. The nonlinearity is the maximum deviation from the dashed line. This is called endpoint nonlinearity, which is defined in the work of Nyce et al. [11]. With a displacement in the range from 30 to 80 µm, the nonlinearity is 0.734 µm. This corresponds to 1.47% of the relative displacement of 50 µm.

## 4. Guard Ring Electrodes

To reduce the slope error and nonlinearity, guard ring electrodes, based on the principle devised by Lord Kelvin and Clerk Maxwell, are used for capacitive displacement measurements. The working electrodes are enclosed by an active shielding, the guard ring. The guard ring has the same electric potential as the corresponding working electrode and is electrically separated from the working electrode by a dielectric gap [12].

**Figure 5:**
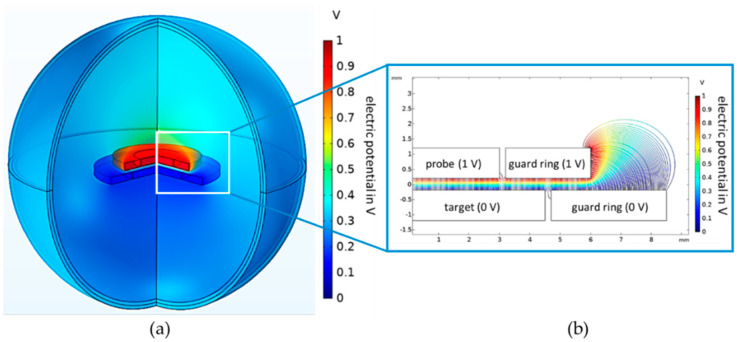
FEM simulation result of the parallel plate capacitor with guard ring electrodes. (**a**) electric potential distribution of the complete model. (**b**) propagation of the electric field lines. There are fringe fields at the edges of the guard rings. The distortions of the field lines at the edges of the working electrodes are much smaller.

The result of the extended simulation model is illustrated in the left part of Figure 5. The guard ring of the probe electrode has a radius of 6 mm, and the guard ring of the target electrode, a radius of 8.5 mm. In this model, air gaps with a width of 40 µm and a relative permittivity of 1 separate the guard rings and the working electrodes.

The calculated capacitance for a distance of 30 µm is

(4) $$C_{simul,\;with\;guard\;ring}=8.47\;\mathrm{pF}.$$

Therefore, the simulated capacitance of a sensor with a guard ring is only 1.6% larger than the capacitance of an ideal parallel plate capacitor.

The distribution of the electric field lines is shown in the right part of Figure 5. At the edges of the guard rings, the electric field lines are strongly disturbed, such as in the case of the simple plate capacitor. The field lines at the edges of the working electrodes can only spread out within the dielectric gaps. This reduces the active area of the probe, and the difference between the active area and the geometric area decreases. This is the reason for the smaller difference between the simulated capacitance of a sensor with a guard ring and the capacitance of an ideal parallel plate capacitor. Furthermore, the distortion of the field lines at the edges of the electrodes is significantly smaller compared to the distortion at the edges of the guard rings.

**Figure 6:**
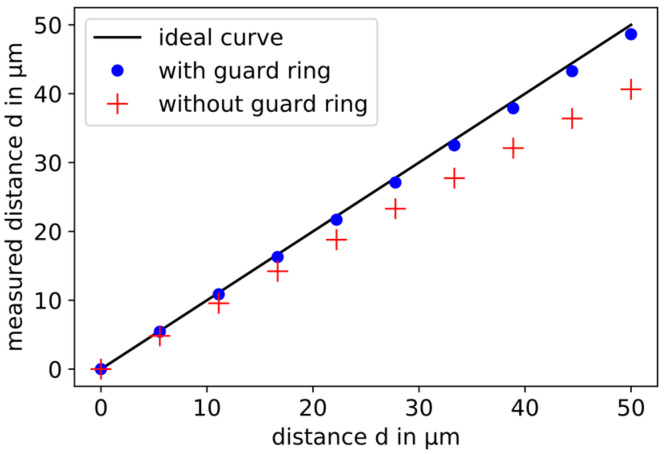
Comparison of the FEM simulation results of capacitive displacement sensors with (blue dots) and without (red crosses) guard rings with the ideal curve (black line). The slope error is reduced by using a guard ring.

The results of the FEM simulation of capacitive displacement measurements with sensors with and without guard rings are plotted together with the ideal curve in Figure 6. The slope error is significantly smaller for a sensor with a guard ring, because the sensitivity of the inverse capacitance is larger with the change in plate distance. The reason is the smaller difference between the active sensor area and the geometric sensor area compared to a sensor without guard ring. In this model, the slope error is 1.37 µm.

**Figure 7:**
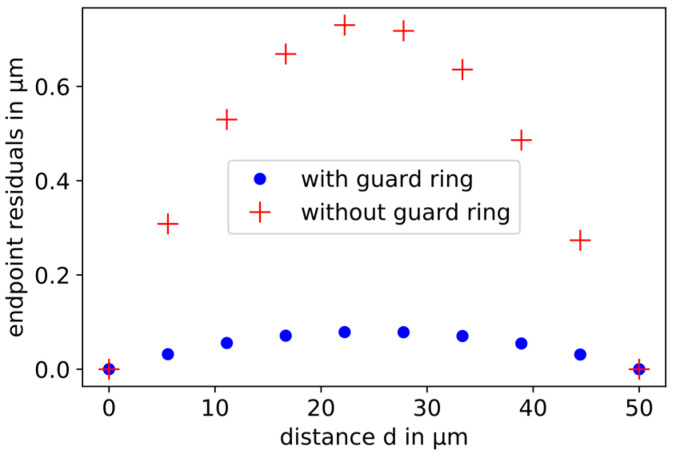
Comparison of the residuals of the simulated capacitive displacement measurement with (blue dots) and without (red crosses) guard rings. Due to the reduction in the distortion of the electric field lines at the edges of the working electrodes, the guard rings lead to a smaller nonlinearity.

In Figure 7, the residuals of the results of the simulated capacitive displacement measurement with and without guard rings are compared. The capacitive sensor with guard ring electrodes has a significantly smaller nonlinearity than the simple parallel plate capacitor, because of the reduction in disturbance of the electric field lines at the edges of the active sensor electrode. The nonlinearity is 0.08 µm. This corresponds to 0.16% of the relative displacement of 50 µm.

**Figure 8:**
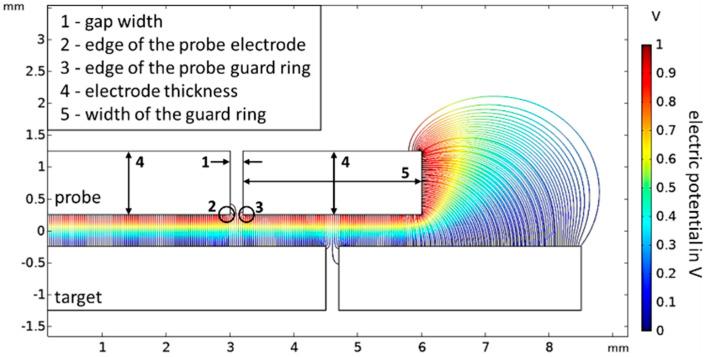
Presentation of the five parameters whose influence on the capacitance, the slope error, and nonlinearity are simulated. The parameters are (1) the width of the dielectric gap, (2) the edge radius of the probe electrode, (3) the edge radius of the probe guard ring, (4) the thickness of the electrodes, and (5) the width of the guard ring.

This guard ring model is the starting point for the following parameter study. In Figure 8, the investigated influencing parameters are summarized. In the next section, results of the simulation of the influence of:

- The width of the dielectric gap;
- The edge radius;
- The thickness of the probe electrode;
- The width of the guard ring,

on the capacitance, the slope error, and the nonlinearity are presented.

## 5. Parameter Study

For the investigation of each individual parameter, 10 displacement measurements with different sensor geometries are simulated. For each simulated displacement measurement, only one parameter is changed, while the others are kept constant. The simulated capacitances, slope errors, and nonlinearities are plotted against the respective sensor parameters.

The dependence of the influences of the geometric sensor properties on the measuring range and the starting distance is important for the design of the planned experiment with real capacitive sensors. For this reason, the relative displacement width and the starting distance are varied in the following section as well.

### 5.1. Geometric Sensor Properties

**Figure 9:**
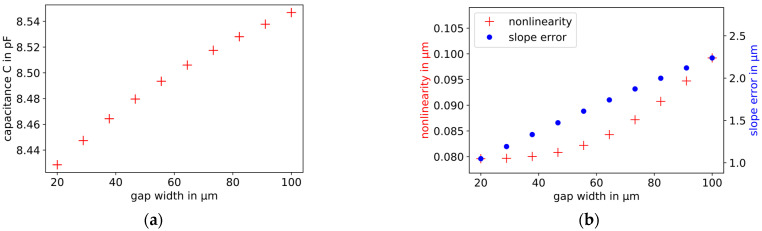
Influence of the width of the gap between the probe electrode and its guard ring on the capacitance (**a**), plate distance = 30 µm), and the slope error (**b**), blue dots, right scale) and nonlinearity (**b**, red crosses, left scale) of capacitive displacement measurements. With increasing gap width, the slope error increases linearly and the nonlinearity exponentially.

The width of the dielectric gap of the probe electrode varies in the range from 20 to 100 µm. With a larger width of the dielectric gap, the capacitance, the slope error, and the nonlinearity increase. The slope error increases linearly, the nonlinearity exponentially. The influence of the gap width on the slope error is approximately 80 times larger in the selected range than on the nonlinearity. The simulation results are shown in Figure 9.

**Figure 10:**
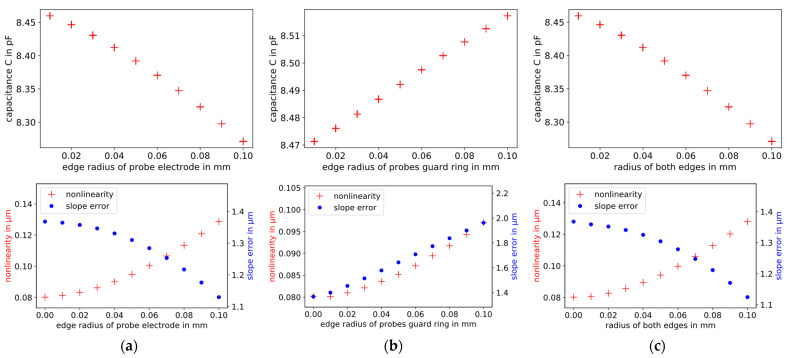
Influence of the edge radius on the capacitance (top, plate distance = 30 µm), the slope error (bottom, blue dots, right scale), and nonlinearity (bottom, red crosses, left scale) of capacitive displacement measurements. The edge radius varies in the range from 0 to 0.1 mm. Three scenarios are simulated: (**a**) changing the edge radius of the probe electrode; (**b**) changing the edge radius of the probe guard ring; (**c**) changing both edge radii.

The influence of the edge radius on the capacitance, the slope error, and nonlinearity is investigated. The edge radius varies in the range from 0 to 0.1 mm. Three scenarios are simulated and shown in Figure 10:

- The edge radius of the probe electrode is increased (Figure 10a);
- The edge radius of the guard ring of the probe electrode is increased (Figure 10b);
- The edge radius of the probe and the edge radius of the probe guard ring are both increased (Figure 10c).

As the edge radius of the probe electrode increases (Figure 10a,c), the capacitance and the slope error decrease, and the nonlinearity increases. As the edge radius changes, the slope error decreases approximately 20 times faster than the nonlinearity increases. With increasing edge radius of the probe guard ring (Figure 10b), the capacitance, the slope error, and the nonlinearity increase. In this case, the influence of the edge radius on the slope error is approximately 40 times larger in the selected range than on the nonlinearity.

**Figure 11:**
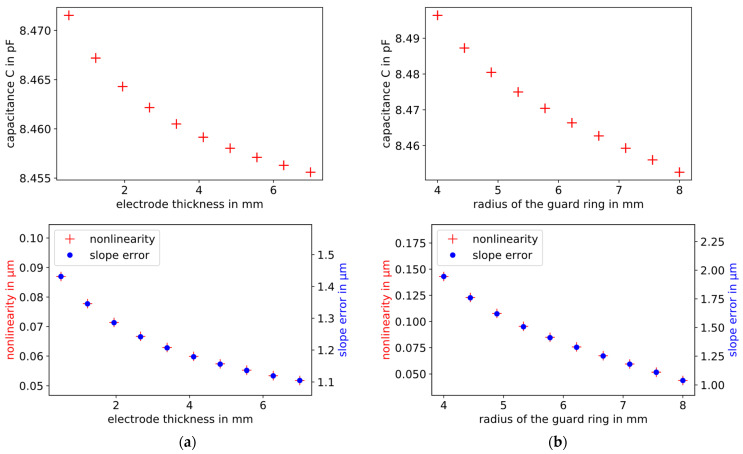
FEM simulation results of the influence of (**a**) the electrode thickness and (**b**) the width of the guard ring of the probe on the capacitance (top, plate distance = 30 µm), and the slope error (blue dots, right scale) and nonlinearity (red crosses, left scale) of capacitive displacement measurements. Due to a thicker electrode and a wider guard ring, the capacitance, the slope error, and the nonlinearity decrease.

The thickness of the electrodes varies in the range from 0.5 to 7 mm, and the width of the guard ring of the probe electrode in the range from 4 to 8 mm. The simulation results are plotted in Figure 11.

The thicker the electrodes and the larger the radius of the guard ring, the smaller the capacitance, the slope error, and the nonlinearity are. In both cases, the influence of the respective parameter on the slope error is approximately 10 times larger than on the nonlinearity.

### 5.2. Measurement Range

Based on the FEM model, an experiment will be designed to verify the simulation results. The expected effects should be made as large as possible to reduce the requirements for the experimental setup. For this purpose, there is a simulation showing how the influence of the guard ring width on the slope error and on the nonlinearity changes for different relative displacement widths and starting distances between the electrodes.

**Figure 12:**
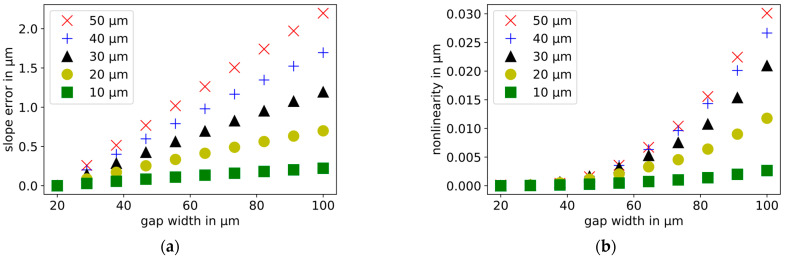
Influence of the relative displacement range on the influence of the width of the dielectric gap on (**a**) the normalized slope error and (**b**) the normalized nonlinearity. The further the relative displacement, the faster the slope error and nonlinearity increase with the increasing dielectric gap.

As shown in Figure 9, displacement measurements are simulated with dielectric gap widths ranging from 20 to 100 µm. The starting distance of each simulation is 30 µm. These simulations are repeated for different relative displacement widths in the range from 10 to 50 µm. In Figure 12, the normalized slope errors and nonlinearities are plotted. The width of the gap has an increasing influence on the slope error and the nonlinearity, respectively, as the displacement range increases.

**Figure 13:**
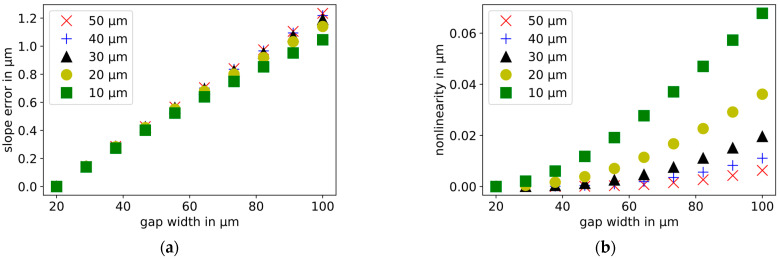
Influence of the starting distance between probe and target on the influence of the width of the dielectric gap on (**a**) the normalized slope error and (**b**) the normalized nonlinearity. The larger the starting distance, the faster the slope error increases and the slower the nonlinearity increases with the increasing dielectric gap width.

In order to investigate the influence of the starting distance on the slope error and the nonlinearity, displacement measurements with different gap widths are repeated. In this case, the relative displacement is 50 µm in each simulation. The starting distances between the probe and the target are varied in the range from 10 to 50 µm. In Figure 13, the normalized slope errors and nonlinearities are presented. The larger the starting distance, the faster the slope error increases and the slower the nonlinearity increases with an increasing dielectric gap width. These simulation results must be considered in the design of the experiment.

## 6. Discussion

In this study, FEM is used to investigate the influence of geometric sensor properties on the capacitance and the slope error and nonlinearity of capacitive displacement measurements. The simulations demonstrate how much the capacitance depends on the distribution of the electric field lines in space. In the Equation (1) of an ideal parallel plate capacitor, only the geometric area is included. In a real capacitive sensor, the field lines extend beyond the geometric sensor surface. Thus, the side surface and the back of the sensor also contribute to the capacitance as stray capacitances. Furthermore, the distribution of the field lines in space results in an active electric sensor area, which is accordingly larger than the geometric sensor area. For these reasons, the simulated capacitance of a simple parallel plate capacitor is larger than the capacitance of an ideal parallel capacitor, as more charge can be stored in the capacitor at a given potential difference.

An active guard ring limits the propagation of the electric field lines. This reduces the difference between the geometric sensor area and the active sensor area. Thus, the difference between the capacitance of an ideal parallel plate capacitor and the simulated capacitance decreases. The slope error resulting from the calculation of the difference between the electrode distance and the “measured” displacement using the ideal Equation (1) is thereby reduced.

In an ideal parallel plate capacitor, the electric field lines propagate parallel to each other between the probe and the target without any distortion. In a real capacitive sensor, the field lines are distorted at the edges. The endpoint nonlinearity of a capacitive displacement measurement depends on how many electric field lines are distorted and by how much. This knowledge will now be used to discuss the results of the parameter study.

**Figure 14:**
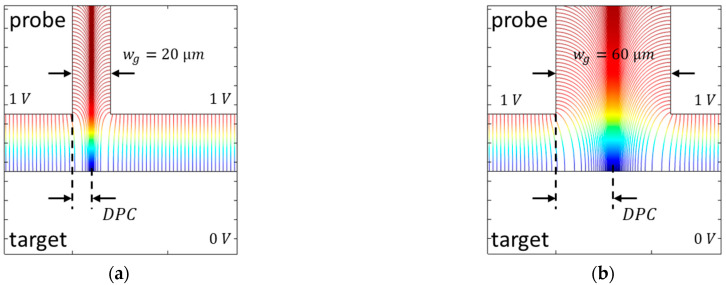
Comparison of the propagation of the electric field lines in the gap between the probe and its guard ring for (**a**) a small gap width and (**b**) a large gap width. The field lines of the side surfaces of the probe and the guard ring converge in the middle of the gap. For wider gap widths, the distance between the probe and the center of the converging field lines (DPC) becomes larger, and the distortion of the electric field lines increases.

The first step was the simulation of the influence of the width of the dielectric gap. With increasing gap width, the capacitance, the slope error, and the nonlinearity increase. The distribution of the electric field lines in the gap between the probe and its guard ring for gap widths of$\;w_{g}=20\;µm$ and$\;w_{g}=60\;µm$ is shown in Figure 14. The distance between the probe and the target is 30 µm.

The field lines of the respective side surface of the probe and its guard ring converge in the middle of the gap, which is consistent with the end of the active electrode in Maxwell’s calculations [12]. With an increasing gap width, the distance between the probe and the center of the converging electric field lines in the gap (DPC) increases. Thus, the active area increases, and the capacitance becomes larger. With an increasing active area, the sensitivity of the inverse capacitance related to the change in electrode distance decreases, and the slope error also becomes larger. The electric field lines are also distorted more with an increasing gap width, and the nonlinearity increases as well.

In the next step, the influence of edge rounding has been simulated. Three different cases have been presented:

1. The edge radius of the probe electrode is increased;
2. The edge radius of the guard ring of the probe electrode is increased;
3. The edge radius of the probe and the edge radius of the probe guard ring are both increased.

The capacitance and the slope error decrease, and the nonlinearity increases with the growing edge radius of the probe electrode. If only the edge radius of the guard ring of the probe electrode is increased, all three variables increase. If both edge radii are enlarged, the simulation result is almost identical to the first case.

**Figure 15:**
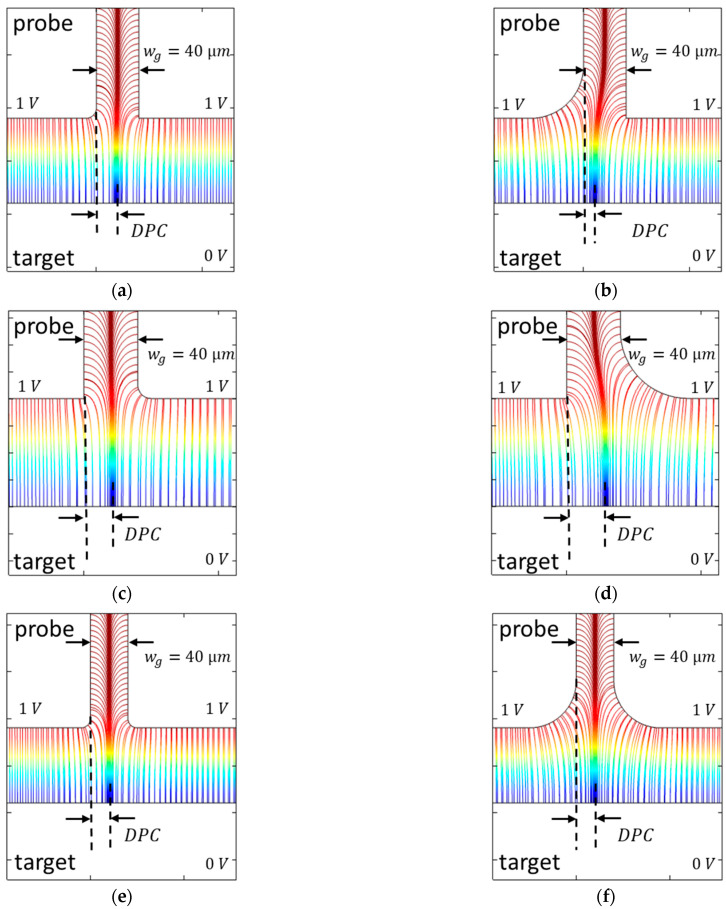
Comparison of the propagation of the electric field lines in the gap between the probe and its guard ring for different edge radii. Three scenarios are presented: (**a**,**b**) the edge radius of the probe electrode is varied; (**c**,**d**) the edge radius of the guard ring of the probe electrode is varied; (**e**,**f**) the edge radius of the probe electrode and the edge radius of its guard ring are both varied. Depending on the edge radius, the field lines are distorted differently. This changes the DPC. Furthermore, in the first and third case, the geometric surface of the probe changes as well.

The distribution of the electric field lines in the gap between the probe and its guard ring is shown in Figure 15. In the three scenarios, the edge radius on the left is $r_{edge}$*=* 0.2 mm, and on the right, $r_{edge}$*=* 0.8 mm. The distance between the probe and the target is 80 µm, and the gap width is 40 µm.

In the first case (Figure 15a,b) the electric field lines are increasingly drawn in the direction of the probe as the edge radius increases. This reduces the DPC. At the same time, the geometric area of the probe electrode is reduced with the increasing edge radius. In the second case (Figure 15c,d) the field lines are distorted with the increasing edge radius in the direction of the guard ring, and the DPC increases. In this case, the surface of the probe electrode is not changed. In the last symmetrical case (Figure 15e,f) the DPC does not change. However, the area of the probe electrode is reduced as in the first case.

In the first and third case, the geometric area of the probe electrode becomes smaller with the increasing edge radius, and the difference between the ideal geometric surface and the active surface decreases. Thus, the capacitance and the slope error decrease as well. Since the simulation results are nearly the same, it can be concluded that the influence of the reduction in the geometric area of the probe electrode with the increasing edge radius is much larger than the change in the DPC. If only the edge radius of the guard ring of the probe electrode is increased, only the DPC and, thus, the capacitance and the slope error increase analogously to the increase in the gap width in Figure 14. In all three scenarios, with the increasing edge radius, the electric field lines are more distorted. Therefore, the nonlinearity increases in all three cases.

**Figure 16:**
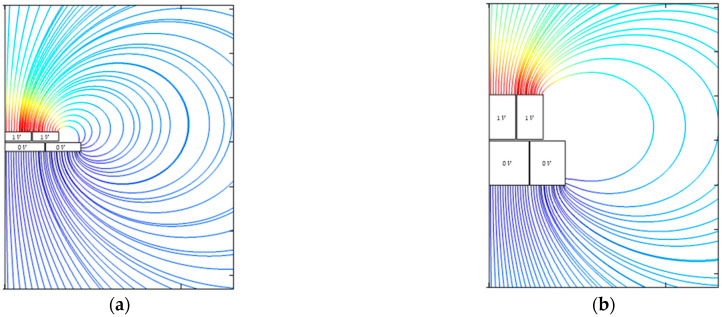
Propagation of the electric field lines outside the capacitor for (**a**) thin electrodes and (**b**) thick electrodes. The thicker the electrodes, the more field lines propagate into the electric far field in a less disturbed manner, and the smaller the top stray capacitance becomes.

**Figure 17:**
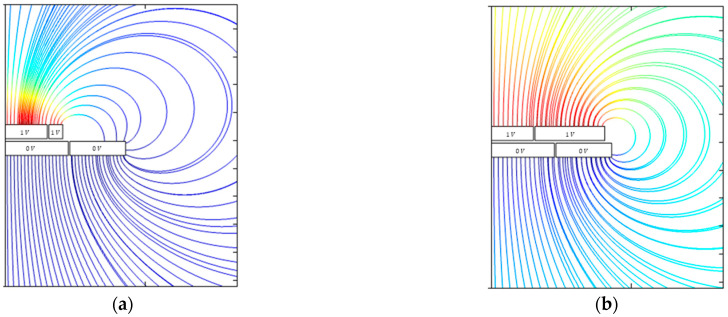
Propagation of the electric field lines outside the capacitor for (**a**) a small probe guard ring and (**b**) a large probe guard ring. The wider the guard ring, the more field lines propagate into the electric far field in a less disturbed way, and the smaller the top stray capacitance becomes.

The influence of the electrode thickness and the width of the guard ring are similar. The larger one of these parameters is, the smaller the capacitance, the slope error, and the nonlinearity are. The distribution of the electric field lines in dependence of the electrode thickness and the width of the guard ring is presented in Figure 16 and Figure 17.

In both cases, there is almost no influence on the distribution of the electric field lines in the gap. However, a change in the thickness of the electrode or the width of the guard ring changes the stray capacitances. Based on Figure 1, the total capacitance of a parallel plate capacitor is

(5) $$C_{total}=C_{ideal}+C_{top}+C_{side}$$

As the electrode becomes thicker or the guard ring wider, the path of the electric field lines from the top of the probe to the target becomes longer, and thus, $C_{top}\;$decreases. As a result, the total capacitance and the slope error decrease. Simultaneously, the electric field lines of the top stray capacitance are less distorted, and the nonlinearity also becomes smaller.

Based on the simulation results, the quantitative magnitude of the individual parameters on the slope error and nonlinearity can be calculated. For this purpose, a manufacturing tolerance of ±10 µm is assumed for each parameter. This means that the change in slope error and nonlinearity are calculated for the case that the gap width, electrode thickness, and guard ring width of the base model vary by ±10 µm. A value of 0.02 mm is selected as the initial value for the edge radius of the probe electrode. The results are listed in Table 1 and Table 2.

With the assumed manufacturing tolerance, the width of the dielectric gap has the largest influence on the slope error. Based on a slope error of 1.37 µm, the percentage change for the assumed manufacturing tolerance is approximately ±12%.

The change in the radius of the edge of the probe electrode has the largest influence on nonlinearity. The nonlinearity of the initial model is 0.08 µm. For the assumed manufacturing tolerance of −10 µm, the percentage change in nonlinearity is 2.4%, and for the assumed manufacturing tolerance of +10 µm, the percentage change in nonlinearity is 3.7%.

In all cases, the quantitative influence of the respective parameter change on the slope error is larger than on the nonlinearity. Furthermore, the slope error and the nonlinearity correlate with each other. In Table 3, the Pearson correlation coefficients for the slope error and the nonlinearity for each sensor parameter are listed.

To approve the simulated results according to the influences of the sensor parameters, we recommended enlarging the shown effects. Since the slope error is the dominant one, we will initially focus our experiments on it. By measuring the nonlinearity, we can investigate whether the correlation between the two quantities can be experimentally proven.

The simulation results show that the displacement range and the starting distance affect the influence of the sensor parameters on the slope error and nonlinearity. Therefore, the effects can be increased by selecting the appropriate measuring range. For comparable and straightforward measurements, both must be kept constant. The displacement width should be as large as possible, and the starting distance between the electrodes should be as small as possible to facilitate the experimental investigation of the influence of the gap width on the capacitive displacement measurement.

In real displacement measurements with capacitive sensors, there are additional parameters that influence the slope error and nonlinearity. For example, the surface of a real sensor has a form deviation and is not an equipotential surface [25,26]. Furthermore, in real displacement measurements, the probe and target can never be perfectly aligned to each other. The tilt is one of the major factors influencing the slope error and nonlinearity and has not been considered in the model presented. Moreover, environmental influences and the properties of the measurement electronics must be taken into account in real displacement measurements. The investigation of the influence of these additional factors will be included in our future work.

## 7. Conclusions

The purpose of this project is to obtain a better understanding of the parameters influencing the slope error and nonlinearity of capacitive displacement measurements. In this study, an FEM model was developed, which represents the geometry of real capacitive sensors.

In the first section, we compared the ideal electrical capacitance of a parallel plate capacitor with the electrical capacitance calculated by the FEM. In contrast to the ideal model, stray capacitances and fringe fields are considered in the FEM simulation. The simulated capacitance is 7.6% larger than the capacitance of an ideal parallel plate capacitor.

The FEM model is used to calculate the electrical capacitances for 10 plate distances in the range from 30 to 80 µm. The electrical capacitances are converted into plate distances using the ideal Equation (1). This corresponds to the simulation of a displacement measurement. The slope error as the difference between the maximum calculated displacement and the actual maximum plate distance as well as the endpoint nonlinearity are calculated.

In the next step, we were able to show the improvement of the sensor by using guard ring electrodes. This active shielding limits the propagation of the electric field lines. Thus, the difference between the geometric area and the active area of the probe electrode is reduced. As a result, the slope error is reduced from 9.36 to 1.37 µm. Due to a minor disturbance of the electric field lines, the nonlinearity is reduced from 730 to 80 nm.

This guard ring electrode model is the basis of a parameter study in which the influence of the following sensor parameters on the capacitance, the slope error, and the nonlinearity are investigated:

- The width of the dielectric gap;
- The radius of the edges;
- The thickness of the probe electrode;
- The width of the guard ring.

Based on the characteristics of the field lines, the following conclusions can be summarized:

1. In the case of symmetrical edges, the electric field lines of the side surfaces of the probe electrode and its guard ring converge in the middle of the gap. This is approximately the end of the active surface area.
2. As the gap between the probe electrode and its guard ring increases, the active area also increases. The difference between the ideal geometric probe area and the active probe area becomes larger.
3. As the edge radius of the probe electrode increases, the geometric surface of the probe electrode decreases. The difference between the ideal geometric area and the active area becomes smaller.
4. By increasing the guard ring edge radius of the probe electrode, the field lines are distorted in the direction of the guard ring. The difference between the ideal geometric area and the active area increases.
5. A change in the electrode thickness and the width of the guard ring influences the electric field outside the electrodes.
6. The larger the difference between the ideal geometric area and the active area, the larger the slope error.
7. The more electric field lines are distorted and the stronger this distortion, the larger the nonlinearity.

Therefore, for a small slope error and small nonlinearity, the gap width must be minimal, the edges of the probe electrode and its guard ring must be sharp, the electrodes must be thick, and the guard ring must be wide.

For an assumed manufacturing tolerance of ±10 µm, the changes in slope error and nonlinearity were calculated for the respective parameter change. The width of the dielectric gap has the largest influence on the slope error among the parameters considered. If the width of the dielectric gap between the probe electrode and its guard ring changes within a range of ±10 µm, the slope error changes by ±12%.

The radius of the edge of the probe electrode has the largest influence on the nonlinearity among the parameters considered. If the edge radius changes from 0.2 mm by minus 10 µm, the nonlinearity changes by minus 2.4%. If the edge radius changes by plus 10 µm, the nonlinearity changes by plus 3.7%. With this knowledge, it can be determined if an improvement in the capacitive displacement sensor manufacturing process is necessary under the given manufacturing tolerances.

Furthermore, the parameter study shows that the influence of individual parameters on the slope error is larger than on the nonlinearity. Moreover, simulations show that the slope error and the nonlinearity correlate for all parameters.

In addition, we have simulated how the influence of the gap width on the slope error and nonlinearity changes depending on displacement width and starting distance. It could be shown that a wide displacement with a short starting distance is useful for investigating the influence of gap width on displacement measurement.

The results of the parameter study are being used to design an experiment. Capacitive sensors with different gap widths were manufactured using ultra-precision diamond turning. Capacitive displacement measurements are being performed, and the slope error and nonlinearity are being measured as a function of the gap width. In a future publication, the experimental results will be compared with the results of this study.

The FEM model will be extended in the next step. A surface form deviation and a surface potential distribution will be added to the sensor model. In a future parameter study, the influence of these surface properties on the slope error and the nonlinearity will be investigated. The results will be used to calculate a comprehensive uncertainty budget and to look for possibilities to further improve capacitive displacement sensors.

## Figures and Tables

**Table 1:** Changes in the slope error depending on the sensor parameters for a given manufacturing tolerance of ±10 µm.

Base Slope Error = 1.37 µm	Slope Error Difference in nm	
Parameter	−10 µm	+10 µm
gap width	−159.02	155.93
electrode thickness	1.09	−1.09
radius of the guard ring	1.85	−1.85
edge radius of the probe	6.93	−11.16

**Table 2:** Changes in the nonlinearity depending on the sensor parameters for a given manufacturing tolerance of ±10 µm.

Base Nonlinearity = 80 nm	Nonlinearity Difference in nm	
Parameter	−10 µm	+10 µm
gap width	−0.44	1.08
electrode thickness	0.12	−0.12
radius of the guard ring	0.20	−0.20
edge radius of the probe	−1.94	2.94

**Table 3:** Pearson correlation coefficients of slope error and nonlinearity depending on the sensor parameters.

Parameter	Pearson Correlation Coefficient
gap width	0.93
electrode thickness	1.00
radius of the guard ring	1.00
edge radius of the probe	−1.00

## Data Availability

Not applicable.
